# Air Trapping and the Risk of COPD Exacerbation: Analysis From Prospective KOCOSS Cohort

**DOI:** 10.3389/fmed.2022.835069

**Published:** 2022-03-11

**Authors:** Youlim Kim, Sang Hyuk Kim, Chin Kook Rhee, Jae Seung Lee, Chang Youl Lee, Deog Kyeom Kim, Kyeong-Cheol Shin, Ki Suck Jung, Kwang Ha Yoo, Yong Bum Park

**Affiliations:** ^1^Division of Pulmonary, Allergy and Critical Care Medicine, Department of Internal Medicine, Konkuk University Hospital, School of Medicine, Konkuk University, Seoul, South Korea; ^2^Division of Pulmonology and Critical Care Medicine, Department of Medicine, Samsung Medical Center, Sungkyunkwan University School of Medicine, Seoul, South Korea; ^3^Division of Pulmonary, Allergy and Critical Care Medicine, Department of Internal Medicine, Hallym University Kangnam Sacred Heart Hospital, Hallym University College of Medicine, Seoul, South Korea; ^4^Division of Pulmonary and Critical Care Medicine, Department of Internal Medicine, Seoul St. Mary's Hospital, College of Medicine, The Catholic University of Korea, Seoul, South Korea; ^5^Department of Pulmonary and Critical Care Medicine, Asan Medical Center, University of Ulsan College of Medicine, Seoul, South Korea; ^6^Division of Pulmonary, Allergy and Critical Care Medicine, Department of Internal Medicine, Hallym University Chuncheon Sacred Heart Hospital, Chuncheon, South Korea; ^7^Department of Pulmonary, Allergy and Critical Care Medicine, Seoul Metropolitan Government Seoul National University Boramae Medical Center, Seoul, South Korea; ^8^Division of Pulmonology and Allergy, Regional Center for Respiratory Disease, Yeungnam University Medical Center, Daegu, South Korea; ^9^Division of Pulmonary, Allergy and Critical Care Medicine, Department of Internal Medicine, Hallym University Sacred Heart Hospital, Anyang, South Korea; ^10^Division of Pulmonary, Allergy and Critical Care Medicine, Department of Internal Medicine, Hallym Univeristy Kangdong Sacred Heart Hospital, Seoul, South Korea

**Keywords:** chronic obstructive pulmonary disease, lung volume measurements, total lung capacity, exacerbation, air trapping

## Abstract

**Background and Aims:**

Air trapping is a predictive index for a decline in lung function and mortality in patients with chronic obstructive pulmonary disease (COPD). However, the role of air trapping in COPD exacerbation has rarely been studied. Therefore, this study aimed to investigate the impact of air trapping as a continuous parameter on COPD exacerbation.

**Materials and Methods:**

To evaluate air trapping, we identified the ratio of residual volume (RV) to total lung capacity (TLC) of patients with COPD from the Korean COPD Subgroup Study (KOCOSS) cohort, which is a multicenter-based, prospective, consecutive cohort in Korea. The primary outcome was a development of COPD exacerbation during 3 years of follow-up.

**Results:**

Of 2,181 participants, 902 patients measured the RV/TLC ratio in the baseline enrollment, and 410 were evaluated for assessing the development of COPD exacerbation. Of 410 patients, the rate of moderate to severe exacerbation and severe exacerbation was 70.7% and 25.9%. A 10% increase of RV/TLC ratio increased the risk of the moderate to severe exacerbation by 35% and severe exacerbation by 36%. In subgroup analysis, an interaction effect between triple inhaled therapy and the RV/TLC ratio for severe exacerbation nullified the association between the RV/TLC ratio and severe exacerbation (*p* for interaction = 0.002).

**Conclusions:**

In this prospective cohort study, we found that air trapping (representing RV/TLC ratio as a continuous parameter) showed an association with an increased risk of COPD exacerbation, particularly in patients who have not undergone triple inhaler therapy.

## Introduction

The Global Initiative on Obstructive Lung Disease (GOLD) defines Chronic Obstructive Pulmonary Disease (COPD) as a common, preventable, and treatable disease characterized by persistent respiratory symptoms and airflow limitation (1). Despite advances in the strategy for the treatment of COPD, the burdens of COPD are substantial (2, 3). In particular, acute exacerbation is a critical factor worsening the prognosis of patients with COPD (4). Thus, predicting and preventing acute exacerbations is the cornerstone in managing patients with COPD (5). In addition, it is vital to investigate parameters related to the risk of COPD exacerbation and to figure out appropriate interventions.

Air trapping, which is common in chronic respiratory diseases, occurs when the lungs become abnormally enlarged due to peripheral airway obstruction (1, 6). Air trapping can be quantified by the ratio of residual volume (RV) to total lung capacity (TLC) (7). Several studies have found the clinical significance of the RV/TLC ratio in managing chronic respiratory diseases (8–10). The RV/TLC ratio is also known as a predictive index for various adverse outcomes of COPD, such as a decline in lung function and increased mortality (11–13). However, studies on the association between the RV/TLC ratio and COPD exacerbation are rare.

Although one study found that the RV/TLC ratio was associated with COPD exacerbation, it performed the only simple Cox regression analysis and did not assess the severity of exacerbation in–depth (14). Consequently, the relationship between the RV/TLC ratio and COPD exacerbation was not fully understood. It is imperative to gain a deep understanding of the role of the RV/TLC ratio in the management of patients with COPD, particularly for predicting severe exacerbation. Therefore, we aimed to investigate the impact of the RV/TLC ratio on COPD exacerbation, with emphasis on the non-linear association and severity of exacerbation, using data from a multicenter-based prospective, longitudinal cohort study.

## Methods

### Study Population

**Figure 1:**
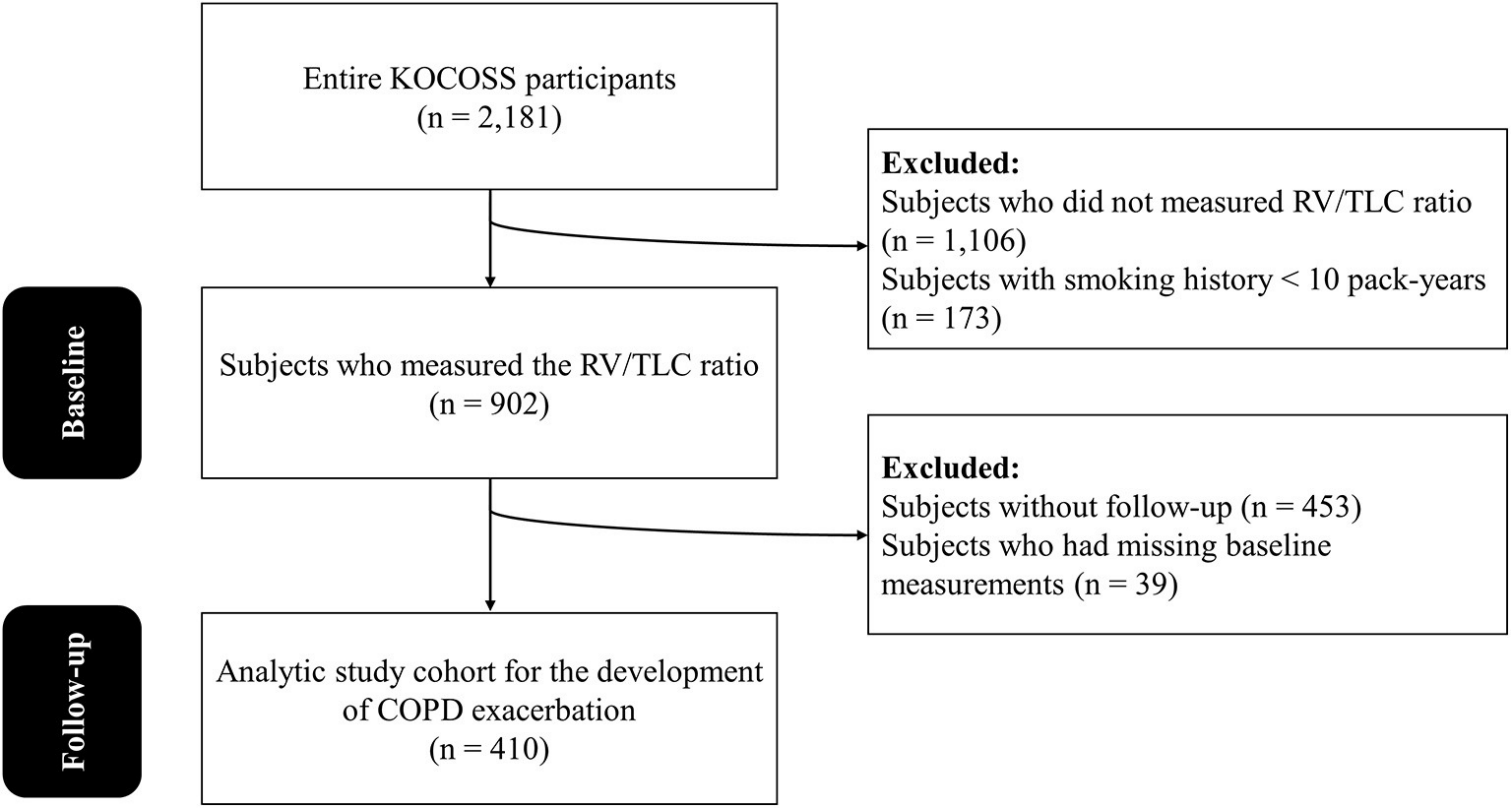
The flow chart of the study participants. KOCOSS, the Korean COPD Subgroup Study; RV, residual volume; TLC, total lung capacity; COPD, chronic obstructive pulmonary disease.

The Korean COPD Subgroup Study (KOCOSS) cohort is a multicenter-based, prospective, consecutive cohort to explore risk factors of COPD progression. Study participants' enrollment and baseline measurements were performed from December 2012 and October 2014. Inclusion criteria were as follows: (1) physician-diagnosed patients with COPD, (2) aged ≥ 40 years, (3) positive respiratory symptoms, and (4) post-bronchodilator ratio of forced expiratory volume in one second (FEV_1_) to forced vital capacity (FVC) <0.7. A previous study has described more detailed information about this cohort (15). First, 2,181 patients were enrolled in entire KOCOSS cohort. Of 2,181 patients, we excluded 1,106 who did not measure RV/TLC ratio and 173 with smoking history <10 pack-years. As results, 902 patients who measured the RV/TLC ratio were included for our study, and baseline characteristics was analyzed. Finally, analyses for assessing the development of COPD exacerbation were performed in 410 patients, excluding 453 with missing follow-up and 39 with missing baseline measurements (Figure 1).

### Exposure: RV/TLC Ratio

The exposure of this study was the RV/TLC ratio. The RV/TLC ratio was measured using body plethysmography (V 6200 [SensorMedics] or PF/DX [MedGraphyics]). The absolute value was used, and there was no additional adjustment following the American Thoracic Society (ATS)/European Respiratory Society (ERS) recommendations (16).

### Outcomes: COPD Exacerbation

The outcome was the development of COPD exacerbation, which was observed prospectively over 3 years. The COPD exacerbation was defined as worsening of respiratory symptoms such as sputum, cough, and dyspnea (1). Moderate exacerbation was defined as an exacerbation requiring systemic steroids or antibiotics but manageable at an outpatient department without hospitalization. Severe exacerbation was defined as an exacerbation requiring emergency room visits or hospitalization.

### Covariates

Body mass index (BMI) was calculated by dividing the weight by the square of the height (kg/m^2^). Occupational exposure was defined as respondents who answered “yes” to the question “Did you work in a dusty or bad air environment?”. Symptoms were assessed using a modified Medical Research Council (mMRC) dyspnea scale and COPD Assessment Test (CAT) score (17, 18). Pre-bronchodilator and post-bronchodilator FVC, FEV_1_, and diffuse capacity for carbon monoxide (DL_CO_) measured using spirometry as recommended by the ATS/ERS (19). The Korean formula was used to calculate the percentages of predicted values of FVC and FEV_1_ (20). We used self-reported inhaler use and patient-reported physician diagnosis for comorbidities. Inhaler use was classified as long-acting beta-agonist (LABA), long-acting muscarine-antagonist (LAMA), inhaled corticosteroid (ICS), ICS/LABA, LABA/LAMA, and LABA/LAMA/ICS (triple inhaled therapy). The choice of inhaler was made at the physician's discretion.

### Ethical Considerations

This study was conducted according to the Helsinki declaration. Our study protocol was approved by the Ethics Committee of each participating medical center. All data were provided anonymously and all participants provided written informed consent prior to enrollment.

### Statistical Analyses

Continuous variables were expressed as medians with interquartile ranges (IQR), and categorical variables were expressed as numbers with percentages. In the analysis of the p-trend, we used the Jonckheere-Terpstra test for continuous variables and the Cochran-Armitage test for categorical variables. The rate of COPD exacerbation was assessed with the number of exacerbations, by classifying into the 4 IQR groups of the RV/TCL ratio and a density plot was used to analyzed the frequency of COPD exacerbation.

The association between the RV/TLC ratio and the risk of COPD exacerbation was evaluated using logistic regression analysis. In the multivariable model, age, sex, college graduate, occupational exposure, BMI, mMRC, categorized FEV_1_ %-predicted, smoking amount, triple inhaled therapy, cardiovascular disease, hypertension, and diabetes mellitus were included for adjustment. The non-linear relationships between the RV/TLC ratio and the risk of COPD exacerbation were further explored by restricted cubic spline curve analysis adjusted for previously mentioned variables using “rms” package of R. The FEV_1_ %-predicted was categorized by the rounded value of median in the adjustment due to the strong correlation with the RV/TLC ratio (Supplementary Figure S1). To assess the added value of the RV/TLC ratio compared to the FEV_1_ %-predicted, a contingency table for COPD exacerbation according to FEV_1_ %-predicted and RV/TLC ratio was made.

Subgroup analysis was performed on clinically significant variables (age, BMI, smoking amount, occupational exposure, mMRC, FEV_1_ %-predicted, triple inhaled therapy, hypertension, and diabetes mellitus). In the adjustment for subgroup analysis, sex was excluded due to the small portion of the female. Subgroups of continuous variables were divided by the median value at the baseline. To evaluate the effect of triple inhaled therapy on COPD exacerbation, a sensitivity analysis was performed after excluding triple bronchodilator users. A two-sided *p*-value <0.05 was considered statistically significant. We performed all statistical analyses using R version 4.0.3 ((21); R Foundation for Statistical Computing, Vienna, Austria).

## Results

### Baseline Characteristics

Baseline characteristics of the study population are shown in Table 1. Participants included 902 patients; 410 of these patients were evaluated for assessing the development of COPD exacerbation. Of the 902 patients, the median (IQR) of age was 70 (64–74) years, and the proportion of males was 96.7%. Among 410 patients followed up for 3 years, the rate of moderate to severe exacerbation and severe exacerbation was 70.7 and 25.9%.

**Table 1:** Baseline characteristics of the study population.

	**RV/TLC ratio quartiles**				
	**Q1: <34%** **(*n* = 218)**	**Q2: 34–40%** **(*n* = 223)**	**Q3: 41–48%** **(*n* = 223)**	**Q4: ≥49%** **(*n* = 238)**	***P*** **_trend_**
Age (years)	67.0 (60.0–73.0)	68.0 (63.0–73.0)	71.0 (66.0–75.0)^*^^†^	72.0 (66.0–76.0)^*^^†^	<0.001
Male	208 (95.4)	217 (97.3)	218 (97.8)	229 (96.2)	0.620
BMI (kg/m^2^) (*n* = 899)	23.6 (22.1–25.8)	23.4 (21.8–25.7)	22.7 (20.7–25.2)^*^^†^	22.2 (19.9–24.7)^*^^†^^‡^	<0.001
College graduate (*n* = 897)	36 (16.8)	30 (13.5)	20 (9.0)^*^	23 (9.7)^*^	0.009
Occupational exposure (*n* = 885)	77 (36.5)	96 (44.0)	87 (39.7)	99 (41.8)	0.447
**Smoking history**					
Past smoker	148 (67.9)	151 (67.7)	152 (68.2)	178 (74.8)	0.127
Current smoker	70 (32.1)	72 (32.3)	71 (31.8)	60 (25.2)	0.113
Smoking amount (pack-years)	40.0 (28.0–52.0)	40.0 (26.5–51.5)	44.0 (30.0–52.0)	42.0 (26.0–55.0)	0.308
**Symptom assessment**					
mMRC ≥ 2 (*n* = 900)	48 (22.0)	71 (31.8)^*^	71 (31.8)^*^	127 (53.8)^*^^†^^‡^	<0.001
CAT score ≥ 10	141 (64.7)	157 (70.4)	153 (68.6)	199 (83.6)^*^^†^^‡^	<0.001
**Acute exacerbation over 3 years**					
Moderate to severe exacerbation	40 (54.1)	63 (60.0)	77 (68.1)	128 (81.5)^*^^†^^‡^	<0.001
Severe exacerbation	10 (13.5)	12 (11.4)	30 (26.5)^†^	57 (36.3)^*^^†^	<0.001
**Lung function**					
FVC (L)	3.8 (3.3–4.2)	3.5 (3.2–3.9)^*^	3.2 (2.9–3.5)^*^^†^	2.6 (2.3–2.9)^*^^†^^‡^	<0.001
FVC (%-predicted) (*n* = 899)	91.4 (82.1–99.5)	83.5 (75.5–92.2)^*^	77.1 (70.9–85.0)^*^^†^	65.1 (57.4–72.9)^*^^†^^‡^	<0.001
FEV_1_ (L)	2.2 (1.8–2.5)	1.9 (1.6–2.1)^*^	1.6 (1.3–1.9)^*^^†^	1.1 (0.9–1.4)^*^^†^^‡^	<0.001
FEV_1_ (%-predicted) (*n* = 901)	73.9 (61.5–83.6)	63.2 (52.3–71.9)^*^	55.2 (46.6–64.6)^*^^†^	40.7 (31.8–51.1)^*^^†^^‡^	<0.001
FEV_1_/FVC (%)	59.0 (51.0–64.0)	55.0 (46.0–63.0)^*^	51.0 (41.5–59.0)^*^^†^	44.5 (35.0–54.0)^*^^†^^‡^	<0.001
DL_CO_ (%-predicted) (*n* = 882)	67.1 (53.8–78.8)	65.8 (54.0–78.5)	62.9 (50.8–74.5)^*^	57.0 (46.1–68.3)^*^^†^^‡^	<0.001
**Inhaler use (*****n*** **=** **843)**					
LABA	24 (11.8)	22 (10.7)	17 (8.1)	20 (8.8)	0.214
LAMA	80 (39.4)	100 (48.8)	111 (53.1)^*^	142 (62.8)^*^^†^	<0.001
ICS/LABA	60 (29.6)	53 (25.9)	78 (37.3)^†^	137 (60.6)^*^^†^^‡^	<0.001
LABA/LAMA	51 (25.1)	51 (24.9)	41 (19.6)	18 (8.0)^*^^†^^‡^	<0.001
ICS/LABA/LAMA	31 (15.3)	32 (15.6)	53 (25.4)^*^^†^	104 (46.0)^*^^†^^‡^	<0.001
**Comorbidities**					
Cardiovascular disease (*n* = 897)	17 (7.8)	19 (8.6)	19 (8.5)	20 (8.4)	0.841
Hypertension (*n* = 899)	96 (44.2)	93 (41.9)	93 (41.7)	99 (41.8)	0.612
Diabetes mellitus (*n* = 899)	33 (15.2)	48 (21.6)	46 (20.6)	44 (18.6)	0.463

*Data are expresses as medians (interquartile ranges) for continuous variables and numbers (percentages) for categorical variables. The p-trend was analyzed using Jonckheere-Terpstra test for continuous variables and Cochran-Armitage test for categorical variables*.

*, †, and ‡ *mean p <0.05 compared to Q1, Q2, Q3 groups of RV/TLC quartile, respectively. Wilcoxon rank-sum tests for continuous variables and Chi-square tests for categorical variables were used for comparison*.

*RV, residual volume; TLC, total lung capacity; Q, quartile; BMI, body mass index; mMRC, modified medical research council; CAT, the chronic pulmonary obstructive disease assessment test; FVC, forced vital capacity; FEV_1_, forced expiratory volume in 1 second; DL_CO_, diffusion lung capacity for carbon monoxide; LABA, long-acting beta-agonist; LAMA, long-acting muscarine antagonist; ICS, inhaled corticosteroid*.

As the RV/TLC ratio increased, age increased, whereas BMI and educational status decreased. However, male sex, occupational exposure, and smoking history were not associated with the RV/TLC ratio. As the RV/TLC ratio increased, the proportion of symptoms and exacerbation history was increased. Regarding lung function, all variables decreased with the RV/TLC ratio increase. In inhaler use, as the RV/TLC ratio increased, the proportion of LAMA, ICS/LABA, and ICS/LABA/LAMA increased, but the proportion of LABA/LAMA decreased. There were no comorbidities that had an association with the RV/TLC ratio.

### RV/TLC Ratio and Risk of COPD Exacerbation

**Figure 2:**
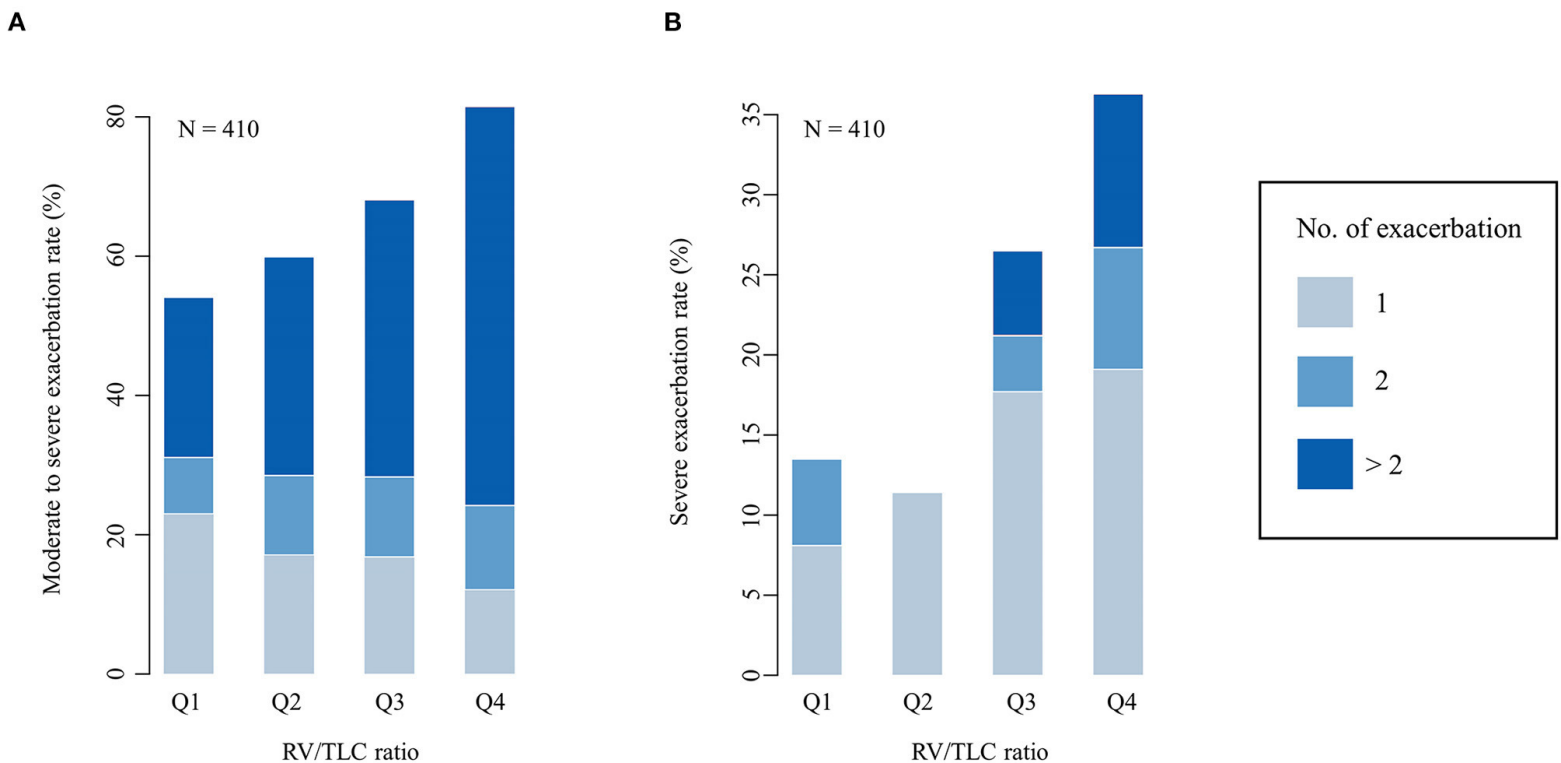
The frequency of COPD exacerbation during 3 years. **(A)** Moderate to severe exacerbation. **(B)** Severe exacerbation. COPD, chronic obstructive pulmonary disease; RV, residual volume; TLC, total lung capacity; Q, quartile.

Over 3 years of follow-up, the rate of moderate to severe exacerbation and severe exacerbation was increased as the RV/TLC ratio increased (Figure 2). This was shown as a similar trend when looking at the frequency of exacerbation (Supplementary Figure S2). This result also observed regardless of FEV_1_ %-predicted, and an increased RV/TLC ratio showed higher rates of COPD exacerbation even in those with preserved FEV_1_ %-predicted (Table 2).

**Table 2:** Incidence of COPD exacerbation during three years according to the percent-predicted FEV_1_ and the RV/TLC ratio.

	Moderate to severe exacerbation			
**N = 410**	**FEV_**1**_: <44%-pred**	**FEV_**1**_: 44–56%**	**FEV_**1**_: 56–69%**	**FEV_**1**_: ≥70%**
	**(n = 122)**	**(n = 121)**	**(n = 102)**	**(n = 65)**
RV/TLC ratio : ≥ 49% (n = 150)	84/94 (89.4)	25/37 (67.6)	11/14 (78.6)	4/5 (80.0)
RV/TLC ratio : 41–48% (n = 104)	17/19 (89.5)	29/41 (70.7)	23/36 (63.9)	3/8 (37.5)
RV/TLC ratio : 34–40% (n = 89)	5/7 (71.4)	21/33 (63.6)	22/34 (64.7)	9/15 (60.0)
RV/TLC ratio : <34% (n = 67)	2/2 (100)	7/10 (70.0)	8/18 (44.4)	20/37 (54.1)
**Severe exacerbation**				
RV/TLC ratio : ≥ 49% (n = 150)	40/94 (42.6)	11/37 (29.7)	5/14 (35.7)	1/5 (20.0)
RV/TLC ratio : 41–48% (n = 104)	7/19 (36.8)	14/41 (34.1)	6/36 (16.7)	1/8 (12.5)
RV/TLC ratio : 34–40% (n = 89)	1/7 (14.3)	4/33 (12.1)	6/34 (17.6)	0/15 (0)
RV/TLC ratio : <34% (n = 67)	0/2 (0)	4/10 (40.0)	2/18 (11.1)	4/37 (10.8)

*Data are expressed as the events/patients (percentages)*.

*Darker shades indicate higher rates of COPD exacerbation*.

*Abbreviations: COPD, chronic obstructive pulmonary disease; FEV_1_, forced expiratory volume in 1 second; RV, residual volume; TLC, total lung capacity*.

In the multivariable analysis, a 10% increase of RV/TLC ratio increased the risk of the moderate to severe exacerbation by 35% and severe exacerbation by 36% (Table 3). Of categorized RV/TLC ratio, the highest quartile of the RV/TLC ratio showed an increased risk of COPD exacerbation compared to the lowest quartile of the RV/TLC ratio [unadjusted odds ratio (OR) 3.87, 95% confidence interval (CI) = 2.05–7.40, *p* < 0.001 for moderate to severe exacerbation; unadjusted OR 3.49, 95% CI 1.71–7.76, *p* < 0.001 for severe exacerbation]. However, these association were nullified after being adjusted for possible confounders (adjusted OR 2.18, 95% CI = 0.99–4.86, *p* = 0.054 for moderate to severe exacerbation; adjusted OR 2.18, 95% CI 0.88–5.73, *p* = 0.102 for severe exacerbation).

**Table 3:** The RV/TLC ratio and the risk of COPD exacerbation.

	**Moderate to severe exacerbation**		**Severe exacerbation**	
***N*** **= 410**	**Unadjusted OR (95% CI, *p*)**	**Adjusted OR (95% CI, *p*)**	**Unadjusted OR (95% CI, *p*)**	**Adjusted OR (95% CI, *p*)**
**RV/TLC ratio**				
Per 10% increase	**1.63 (1.47–2.01**, ** <0.001)**	**1.35 (1.06–1.74, 0.017)**	**1.62 (1.32–2.00**, ** <0.001)**	**1.36 (1.05–1.75, 0.019)**
**RV/TLC ratio quartiles**				
1Q: <34%	1 (ref.)	1 (ref.)	1 (ref.)	1 (ref.)
2Q: 35–40%	1.44 (0.76–2.77, 0.266)	1.20 (0.60–2.44, 0.604)	0.80 (0.32–2.05, 0.643)	0.67 (0.25–1.85, 0.436)
3Q: 41–48%	1.82 (0.97–3.46, 0.064)	1.32 (0.64–2.75, 0.449)	2.10 (0.97–4.87, 0.069)	1.72 (0.70–4.44, 0.243)
4Q: ≥49%	**3.87 (2.05–7.40**, ** <0.001)**	2.18 (0.99–4.86, 0.054)	**3.49 (1.71–7.76, 0.001)**	2.18 (0.88–5.73, 0.102)

*Adjustment for multivariable model: age, sex, college graduate, occupational exposure, BMI, mMRC, categorized FEV_1_ %-predicted, smoking amount, triple inhaled therapy, cardiovascular disease, hypertension, and diabetes mellitus. The bold values indicate statistically significance at the p <0.05 level*.

*COPD, chronic obstructive pulmonary disease; OR, odds ratio; CI, confidence interval; RV, residual volume; TLC, total lung capacity; Q, quartile; BMI, body mass index; mMRC, modified medical research council; FEV_1_, forced expiratory volume in 1 second*.

**Figure 3:**
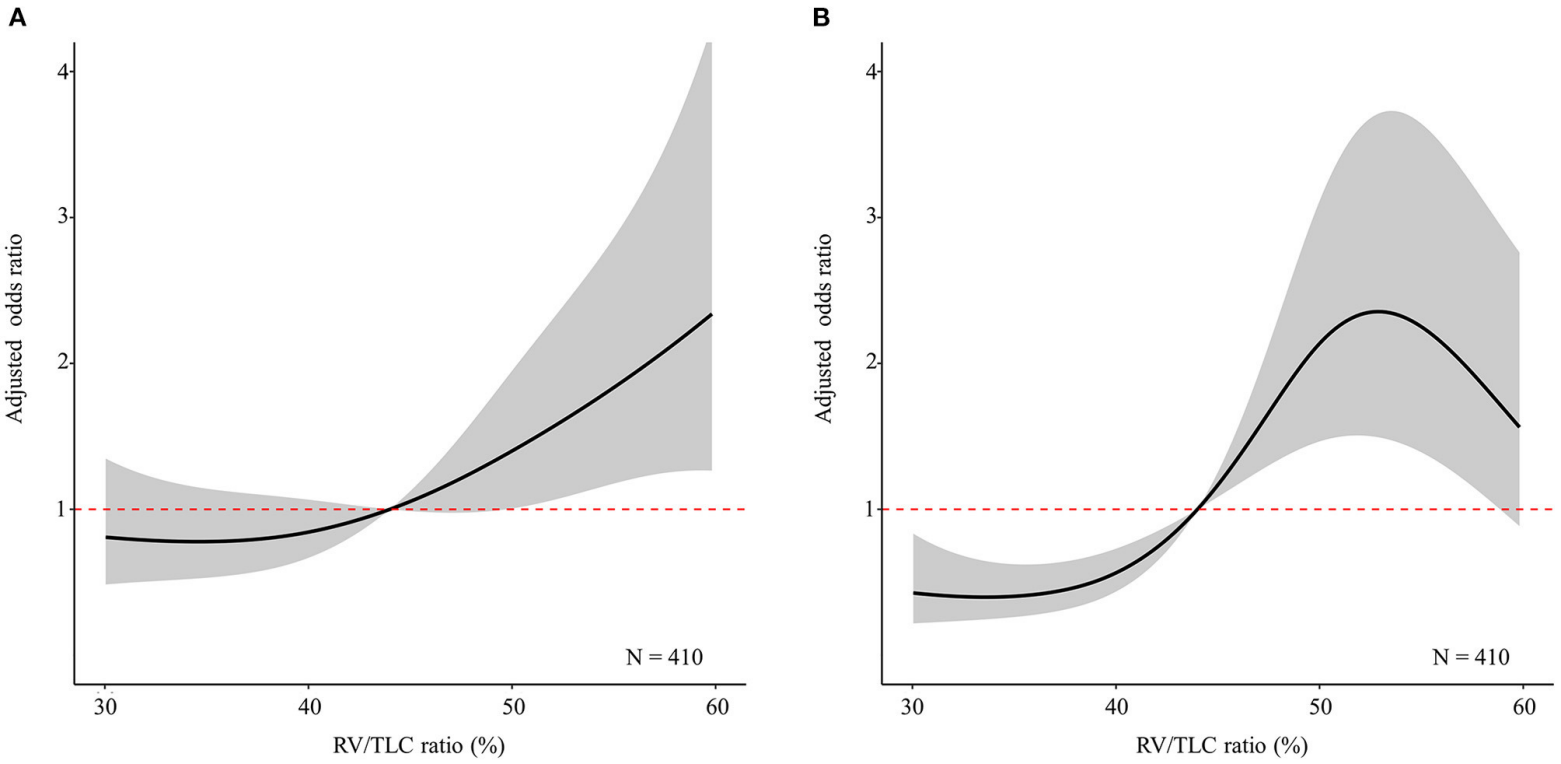
The restricted cubic splines curve of multivariable logistic regression analysis according to the RV/TLC ratio. The solid line indicates the adjusted odds ratio according to the RV/TLC ratio. The shaded region represents the 95% confidence interval for the adjusted odds ratio according to the RV/TLC ratio. **(A)** Moderate to severe exacerbation. **(B)** Severe exacerbation. RV, residual volume; TLC, total lung capacity.

As shown in Figure 3, the relationship between the RV/TLC ratio and moderate to severe exacerbation showed a slow rise. The adjusted OR exceeded one when the RV/TLC ratio was around 45%. Meanwhile, the relationship between RV/TLC ratio and severe exacerbation showed an S-shape curve. To explore factors associated with the S-shaped relationship, we further compared the characteristics in patients with the highest RV/TLC ratio quartile according to the presence or absence of severe exacerbation (Supplementary Table S1). In the patients with the highest quartile of the RV/TLC ratio, those with severe exacerbation history had a lower BMI and FEV_1_/FVC ratio than those without severe exacerbation history.

### Subgroup and Sensitivity Analysis for RV/TLC Ratio and Risk of COPD Exacerbation

In the subgroup analysis, the increase of RV/TLC ratio was associated with an increased risk of moderate to severe exacerbation in the group with age < 70 years, BMI < 23 kg/m^2^, smoking amount ≥40 pack-years, no occupational exposure, no triple inhaled therapy, hypertension, and no diabetes mellitus (Table 4). Meanwhile, in the group with BMI < 23 kg/m^2^, FEV_1_ %-predicted <60%, no triple inhaled therapy, and hypertension, the increase of RV/TLC ratio was associated with an increased the risk of severe exacerbation. The triple inhaled therapy had an interaction effect for the RV/TLC ratio on the risk of severe exacerbation (p for interaction = 0.002). In contrast to the analysis results in triple bronchodilator users, sensitivity analysis showed that an increased RV/TLC ratio was associated with COPD exacerbation in single or dual bronchodilator users (Supplementary Table S2).

**Table 4:** Subgroup analysis of the RV/TLC ratio and the risk of COPD exacerbation.

		**Moderate to severe exacerbation**		**Severe exacerbation**	
**Subgroup**	**No. of patients**	**Adjusted OR (95% CI, *p*)**	***P***_interaction_	**Adjusted OR (95% CI, *p*)**	***P***_interaction_
Age (years)	<70 (*n* = 206)	**1.50 (1.09–2.12, 0.015)**	0.508	1.40 (0.99–1.99, 0.056)	0.958
	≥70 (*n* = 204)	1.08 (0.71–1.64, 0.718)		1.39 (0.95–2.07, 0.097)	
BMI (kg/m^2^)	<23 (*n* = 212)	**1.54 (1.05–2.32, 0.032)**	0.339	**1.49 (1.07–2.12, 0.021)**	0.608
	≥23 (*n* = 198)	1.20 (0.86–1.70, 0.280)		1.16 (0.76–1.76, 0.492)	
Smoking amount (pack-years)	<40 (*n* = 167)	1.21 (0.85–1.75, 0.283)	0.355	1.36 (0.88–2.10, 0.161)	0.867
	≥40 (*n* = 243)	**1.47 (1.04–2.12, 0.032)**		1.35 (0.97–1.88, 0.075)	
Occupational exposure	No (*n* = 233)	**1.47 (1.06–2.05, 0.022)**	0.201	1.43 (1.01–2.03, 0.042)	0.291
	Yes (*n* = 177)	1.15 (0.77–1.72, 0.508)		1.23 (0.82–1.88, 0.320)	
mMRC	<2 (*n* = 238)	1.29 (0.95–1.76, 0.105)	0.377	1.52 (1.02–2.29, 0.041)	0.145
	≥2 (*n* = 172)	1.51 (0.93–2.56, 0.104)		1.22 (0.86–1.76, 0.264)	
FEV_1_ (%-predicted)	<60 (*n* = 142)	1.34 (0.91–2.02, 0.143)	0.696	**2.17 (1.04–4.63, 0.039)**	0.423
	≥60 (*n* = 268)	1.39 (1.00–1.95, 0.052)		1.27 (0.96–1.69, 0.096)	
Triple inhaled therapy	No (*n* = 272)	**1.43 (1.08–1.93, 0.015)**	0.534	**1.79 (1.26–2.56, 0.001)**	**0.002**
	Yes (*n* = 138)	1.11 (0.63–1.98, 0.716)		0.95 (0.64–1.39, 0.781)	
Hypertension	No (*n* = 238)	1.20 (0.86–1.67, 0.276)	0.852	1.13 (0.80–1.59, 0.481)	0.157
	Yes (*n* = 172)	**1.75 (1.17–2.69, 0009)**		**1.70 (1.13–2.65, 0.014)**	
Diabetes	No (*n* = 322)	**1.43 (1.06–1.94, 0.019)**	0.569	1.31 (0.98–1.76, 0.070)	0.935
	Yes (*n* = 88)	1.21 (0.77–1.94, 0.419)		1.42 (0.82–2.46, 0.197)	

*Age, college graduate, occupational exposure, BMI, mMRC, smoking amount, categorized FEV_1_, triple inhaler, cardiovascular disease, hypertension, and diabetes mellitus were used in the adjustment. The bold values indicate statistically significance at the p <0.05 level*.

*RV, residual volume; TLC, total lung capacity; COPD, chronic obstructive pulmonary disease; BMI, body mass index; OR, odds ratio; CI, confidence interval; mMRC, modified medical research council; FEV1, forced expiratory volume in 1 second*.

## Discussion

In this prospective cohort for patients with COPD, we found that the RV/TLC ratio was association with an increased risk of COPD exacerbation, not only moderate to severe exacerbation but also severe exacerbation. There was a significant interaction between triple inhaler therapy and the RV/TLC ratio for severe exacerbation.

Several studies reported an association of air trapping with a poor prognosis of COPD (6, 14, 22, 23). One of those studies reported the RV/TLC ratio was associated with a higher incidence rate of COPD exacerbation (incidence rate ratio = 1.01, 95% CI = 1.00–1.03, *p* = 0.024) (14). However, because the the study focused on the combined evaluation of air trapping and emphysema, there was no additional analysis using the RV/TLC ratio. Similar to the above-mentioned study, our study showed that a 10% increase in the RV/TLC ratio increased the risk of moderate or severe exacerbation by 35%. In addition, we further found that the association between the RV/TLC ratio and COPD exacerbation was significant even when the outcome was confined to severe exacerbations.

As a marker of air trapping, the RV/TLC ratio had a J-shaped relationship with the risk of moderate to severe exacerbation, suggesting a positive correlation with the risk of COPD exacerbation. Recently, several papers have been published that the frequent exacerbation history affects the prognosis or mortality of patients with COPD, and the clinicians are interested in a new concept of “frequent exacerbator phenotype” (24–26). In accordance with a paper describing the frequent exacerbator phenotype (24), multivariable analysis regarding the frequent exacerbation status showed that RV/TLC is the most relevant factor among various variables. Therefore, clinicians should pay attention to measurement of the RV/TLC ratio in patients with COPD at risk of exacerbation.

The S-shaped relationship between the RV/TLC ratio and the risk of severe exacerbation appears likely that other factors may have diminished the need for hospitalization in patients with a very high RV/TLC ratio (27, 28). Another interesting aspect of our study is that the impact of the RV/TLC ratio was nullified after being categorized in multivariable analyses. This finding supports that the RV/TLC ratio may have greater significance as a continuous variable than a categorical variable.

In subgroup analysis, triple inhaler therapy modulated the effect of the RV/TLC ratio on the risk of severe exacerbation. According to the GOLD guidelines, the most favored patients with COPD for triple inhaled therapy are those with severe airflow limitation and frequent exacerbations in spites of dual bronchodilator therapy (1). Several recent studies showed that the triple inhaler significantly reduced the moderate to severe exacerbation (29, 30). Not simply improving exacerbation, they also showed the improvement in lung function and dyspnea symptoms. Considering the pharmacological effect of bronchodilator, which controls the pulmonary hyperinflation while deflating the lung volume, it can be expected to reduce the exacerbation or improve the dyspnea symptom in patients with COPD who had an increased RV/TLC ratio. The interaction between triple inhaled therapy and the RV/TLC ratio was still significant in the analysis in which the eosinophil count was added. In addition, a sensitivity analysis, excluding triple inhaler users, showed that the increased RV/TLC ratio was significantly associated with the risk of COPD exacerbation despite the use of single or dual inhalers. These results would suggest that using a triple inhaler may be an effective intervention to prevent the development of severe exacerbation.

The limitations of our study should be addressed. First, the 3 years of follow-up may not be adequate for assessment of the relationship between the RV/TLC ratio and COPD exacerbation. However, the KOCOSS cohort is an ongoing study, and long-term results will be reported in the future. Second, the loss to follow-up was substantial and may not be random in this cohort, which could lead to selection bias. Third, the number of female patients in the cohort is small because the majority of middle-aged and elderly smokers are male in Korea (31). Fourth, the results of subgroup analysis should be carefully interpreted. In the subgroup analysis, the number of analyzable patients was reduced since the patients were divided into two groups. Therefore, some findings may be only statistical outcomes. Fifth, the details of COPD exacerbation have not been investigated. The RV/TLC ratio may be associated with specific symptoms, such as dyspnea, among various symptoms of exacerbation. Lastly, this study only included patients from the Korean population. Characteristics of COPD may differ according to ethnicity. Thus, the results of our study should be generalized with caution.

Beyond these limitations, our study has strengths. To the best of our knowledge, this is the first prospective study investigating the value of the RV/TLC ratio as a continuous variable using a non-linear analytic method in patients with COPD. In addition, we suggest a possible effective intervention for patient with an increased RV/TLC ratio. We hope that more research will be reported on the clinical significance of the RV/TLC ratio.

In this prospective cohort study, we found an association of air trapping (representing RV/TLC ratio as a continuous parameter) with an increased risk of COPD exacerbation, particularly in patients who have not undergone triple inhaler therapy. This association was still valid for severe exacerbations.

## Data Availability Statement

The raw data supporting the conclusions of this article will be made available by the authors, without undue reservation.

## Ethics Statement

The studies involving human participants were reviewed and approved by the Ethics Committee of each participating medical center. The patients/participants provided their written informed consent to participate in this study.

## Author Contributions

YK: conceptualization, investigation, writing—original draft, and writing—review and editing. SK: conceptualization, methodology, writing—original draft, and writing—review and editing. CR and CL: supervision, validation, and writing—review and editing. JL: investigation, validation, and writing—review and editing. DK: investigation, project administration, and writing—review and editing. K-CS: supervision, project administration, and writing—review and editing. KJ and KY: supervision, funding acquisition, project administration, and writing—review and editing. YP: conceptualization, supervision, validation, and writing—review and editing. All authors approved the final version of the manuscript.

## Funding

This work was supported by the Research Program funded Korea National Institute of Health (Fund CODE 2016ER670100, 2016ER670101, 2016ER670102, 2018ER67100, 2018ER67101, 2018ER67102, and 2021ER120500).

## Conflict of Interest

The authors declare that the research was conducted in the absence of any commercial or financial relationships that could be construed as a potential conflict of interest.

## Publisher's Note

All claims expressed in this article are solely those of the authors and do not necessarily represent those of their affiliated organizations, or those of the publisher, the editors and the reviewers. Any product that may be evaluated in this article, or claim that may be made by its manufacturer, is not guaranteed or endorsed by the publisher.

## Supplementary Material

Supplementary Figure S1 Correlation between the FEV_1_ and the RV/TLC ratio. The strength of correlation was analyzed using Spearman's rank correlation coefficient. FEV_1_, forced expiratory volume in 1 second; RV, residual volume; TLC, total lung capacity.

Supplementary Figure S2 Density plot for the frequency of COPD exacerbation during 3 years. The Red line, yellow line, green line, and blue line indicate the fourth quartile, third quartile, second quartile, and first quartile of the RV/TLC ratio. COPD, chronic obstructive pulmonary disease; RV, residual volume; TLC, total lung capacity.

Supplementary Table S1 Clinical characteristics of patients with the highest RV/TLC quartile according to presence or absence of severe exacerbation.

Supplementary Table S2 Sensitivity analysis for inhaled therapy after the exclusion of triple bronchodilator users.
