# Supplementary figures and images for: Air Trapping and the Risk of COPD Exacerbation: Analysis From Prospective KOCOSS Cohort
Source: Front Med (Lausanne). 2022 Mar 11;9:835069. doi: 10.3389/fmed.2022.835069 (PMC8965692; doi:10.3389/fmed.2022.835069)

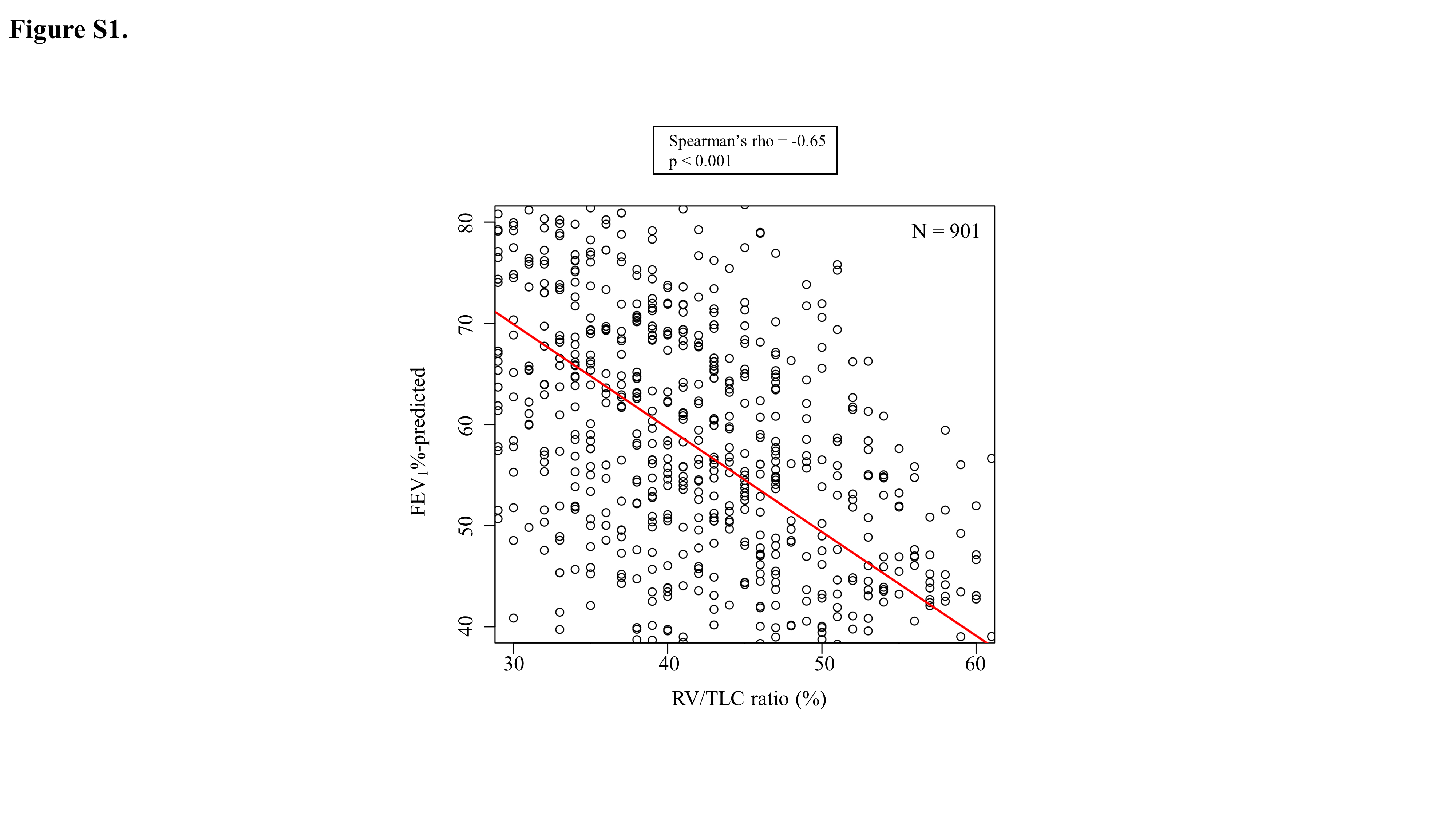

Supplement: Supplementary Figure S1 — Correlation between the FEV1 and the RV/TLC ratio. The strength of correlation was analyzed using Spearman's rank correlation coefficient. FEV1, forced expiratory volume in 1 second; RV, residual volume; TLC, total lung capacity. [file Image_1.TIF]

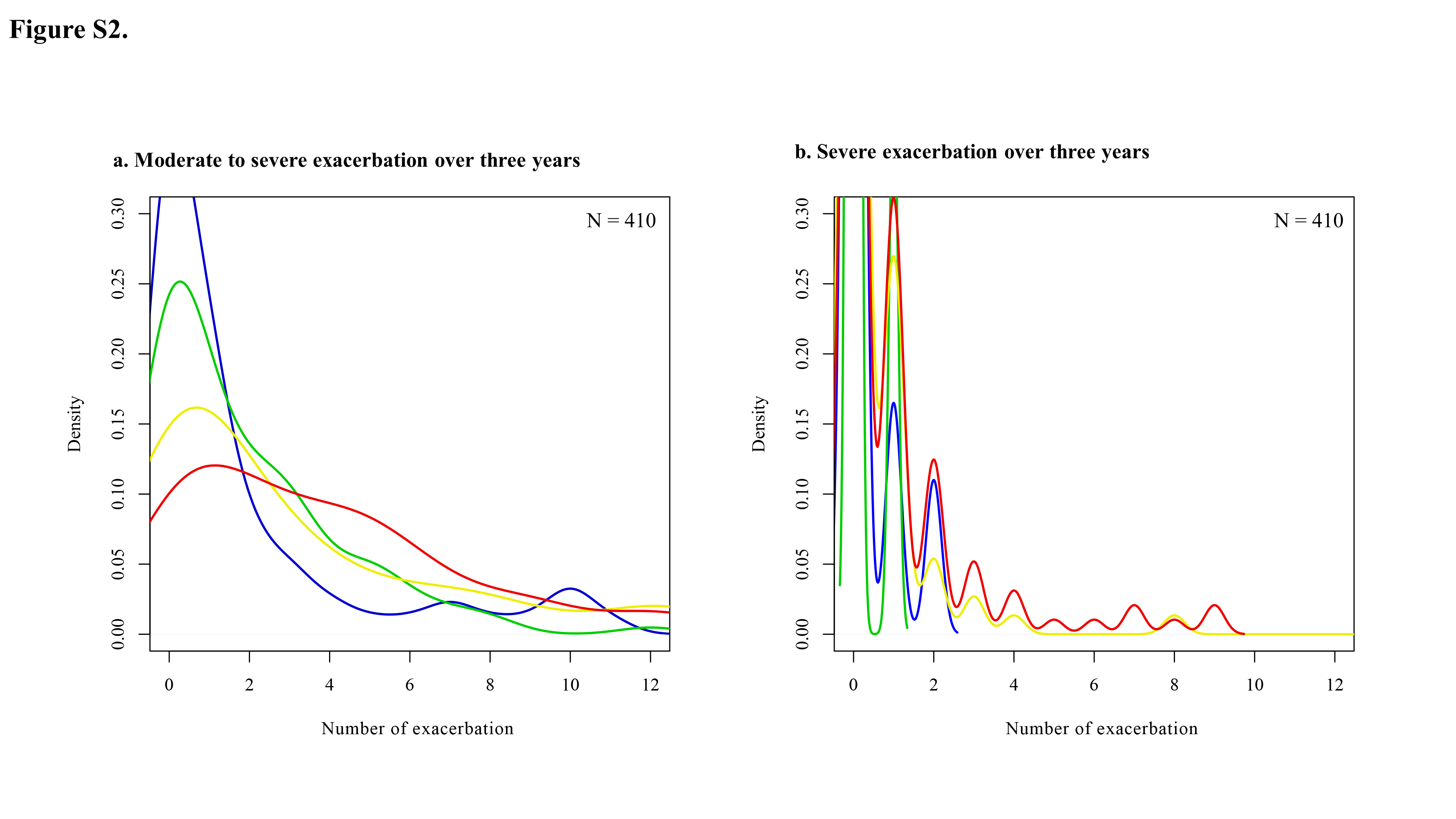

Supplement: Supplementary Figure S2 — Density plot for the frequency of COPD exacerbation during 3 years. The Red line, yellow line, green line, and blue line indicate the fourth quartile, third quartile, second quartile, and first quartile of the RV/TLC ratio. COPD, chronic obstructive pulmonary disease; RV, residual volume; TLC, total lung capacity. [file Image_2.TIF]
