# Supplementary material for: Air Trapping and the Risk of COPD Exacerbation: Analysis From Prospective KOCOSS Cohort
Source: Front Med (Lausanne). 2022 Mar 11;9:835069. doi: 10.3389/fmed.2022.835069 (PMC8965692; doi:10.3389/fmed.2022.835069)
Supplement: Supplementary Table S1 — Clinical characteristics of patients with the highest RV/TLC quartile according to presence or absence of severe exacerbation. [file Table_1.DOCX]

**Table S1. Clinical characteristics of patients with the highest RV/TLC quartile according to presence or absence of severe exacerbation**

| **N = 157** | Without severe exacerbation  (n = 100) | With severe exacerbation  (n = 57) | p-value |
| --- | --- | --- | --- |
| RV/TLC ratio | 0.55 (0.51–0.62) | 0.56 (0.53–0.61) | 0.527 |
| Age (years) | 72 (66–75) | 73 (67–76) | 0.443 |
| Male, n (%) | 97 (97.0) | 54 (94.7) | 0.781 |
| BMI (kg/m^2^) | 22.6 (20.4–24.9) | 20.8 (18.4–23.2) | 0.007 |
| College graduate, n (%) | 8 (8.0) | 7 (12.3) | 0.552 |
| Occupational exposure, n (%) (n = 156) | 43 (43.0) | 22 (38.6) | 0.711 |
| **Smoking history** |  |  | 0.247 |
| Past smoker | 69 (69.0) | 45 (78.9) |  |
| Current smoker | 31 (31.0) | 12 (12.1) |  |
| Smoking amount (pack-years) | 41.5 (25.5–50.5) | 44.0 (34.0–60.0) | 0.195 |
| **Symptom assessment** |  |  |  |
| mMRC ≥ 2 (n = 155) | 47 (47.5) | 37 (64.9) |  |
| CAT score ≥ 10 | 82 (82.0) | 52 (91.2) |  |
| **Lung function** |  |  |  |
| FVC (L) | 2.5 (2.2–2.8) | 2.4 (2.2–2.7) | 0.315 |
| FVC (%-predicted) | 64.3 (55.2–71.5) | 62.0 (57.1–73.5) | 0.916 |
| FEV_1_ (L) | 1.1 (0.9–1.5) | 1.0 (0.8–1.2) | 0.053 |
| FEV_1_ (%-predicted) | 43.1 (33.0–53.0) | 37.0 (31.6–45.5) | 0.106 |
| FEV_1_/FVC (%) | 48.0 (38.0–58.0) | 6.0 (35.0–57.0) | 0.026 |
| DL_CO_ (%-predicted) (n = 146) | 60.2 (48.9–73.9) | 6.6 (44.1–) | 0.186 |
| **Inhaler use (n = 145)** |  |  |  |
| LABA | 12 (12.8) | 6 (10.5) | 0.879 |
| LAMA | 67 (71.3) | 40 (70.2) | 1.000 |
| ICS/LABA | 49 (52.1) | 38 (66.7) | 0.113 |
| LABA/LAMA | 0 (0) | 0 (0) | - |
| ICS/LABA/LAMA | 42 (44.7) | 30 (52.6) | 0.435 |
| **Comorbidities (n = 156)** |  |  |  |
| Cardiovascular disease | 10 (10.0) | 4 (7.0) | 0.734 |
| Hypertension | 41 (41.0) | 26 (45.6) | 0.693 |
| Diabetes mellitus | 24 (24.0) | 9 (15.8) | 0.312 |

Data are expresses as medians (interquartile ranges) for continuous variables and numbers (percentages) for categorical variables. The p-value was calculated using Chi-square test for categorical variables and Wilcoxon rank-sum test for continuous variables.

**Abbreviations:** RV, residual volume; TLC, total lung capacity; BMI, body mass index; mMRC, modified medical research council; CAT, the COPD assessment test; FVC, forced vital capacity; FEV_1_, forced expiratory volume in 1 second; DL_CO_, diffusion lung capacity for carbon monoxide; LABA, long-acting beta-agonist; LAMA, long-acting muscarine antagonist; ICS, inhaled corticosteroid.
