# Supplementary material for: Air Trapping and the Risk of COPD Exacerbation: Analysis From Prospective KOCOSS Cohort
Source: Front Med (Lausanne). 2022 Mar 11;9:835069. doi: 10.3389/fmed.2022.835069 (PMC8965692; doi:10.3389/fmed.2022.835069)
Supplement: Supplementary Table S2 — Sensitivity analysis for inhaled therapy after the exclusion of triple bronchodilator users. [file Table_2.DOCX]

|  |  | Moderate to severe exacerbation | | Severe exacerbation | |
| --- | --- | --- | --- | --- | --- |
|  | No. of patients | Adjusted OR (95% CI, p) | P_interaction_ | Adjusted OR (95% CI, p) | P_interaction_ |
| Inhaled therapy (n = 272) | No (n = 49) | 1.30 (0.54–3.37, 0.570) | 0.740 | 2.79 (0.84–12.30, 0.121) | 0.605 |
|  | Yes (n = 223) | **1.46 (1.07–2.02, 0.020)** |  | **1.69 (1.17–2.47, 0.005)** |  |

**Table S2. Sensitivity analysis for inhaled therapy after the exclusion of triple bronchodilator users**

Inhalation therapy was defined as those who used one or more bronchodilators; dual bronchodialator users (n = 59) and one bronchodilator users (n = 164).

The adjusted OR was calculated after being adjusted for age, college graduate, occupational exposure, BMI, mMRC, categorized FEV_1_ %-predicted, smoking amount, hypertension, and diabetes mellitus.

***Abbreviations:*** OR, odds ratio; CI, confidence interval; BMI, body mass index; mMRC, modified medical research council; FEV_1_, forced expiratory volume in 1 second.
